# Human Sensory, Taste Receptor, and Quantitation Studies on Kaempferol Glycosides Derived from Rapeseed/Canola Protein Isolates

**DOI:** 10.1021/acs.jafc.4c02342

**Published:** 2024-06-18

**Authors:** Christoph Walser, Andrea Spaccasassi, Katrin Gradl, Timo D. Stark, Sonja Sterneder, Frank P. Wolter, Felicia Achatz, Oliver Frank, Veronika Somoza, Thomas Hofmann, Corinna Dawid

**Affiliations:** † Chair of Food Chemistry and Molecular Sensory Science, TUM School of Life Sciences, Technical University of Munich, Lise-Meitner-Str. 34, 85354 Freising, Germany; ‡ Leibniz-Institute for Food Systems Biology at the Technical University of Munich, Lise-Meitner-Str. 34, 85354 Freising, Germany; § TUM School of Life Sciences, Technical University of Munich, Alte Akademie 8a, 85354 Freising, Germany; ∥ Vienna Doctoral School in Chemistry, Faculty of Chemistry, University of Vienna, 1090 Vienna, Austria; ⊥ Department of Physiological Chemistry, Faculty of Chemistry, University of Vienna, 1090 Vienna, Austria; # ZIEL - Institute for Food & Health, Technical University of Munich, 85354 Freising, Germany; ■ Chair of Nutritional Systems Biology, TUM School of Life Sciences, Technical University of Munich, 85354 Freising, Germany; ∇ NPZ Innovation GmbH, 24363 Holtsee, Germany; ○ Professorship for Functional Phytometabolomics, TUM School of Life Sciences, Technical University of Munich, Lise-Meitner-Str. 34, 85354 Freising, Germany

**Keywords:** rapeseed, canola, bitter taste, astringency, alternative protein, kaempferol, kaempferol glucosides

## Abstract

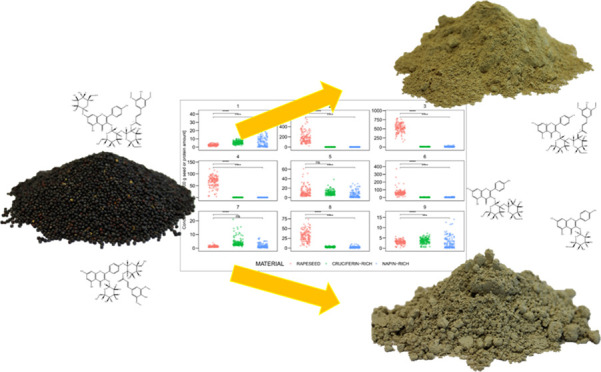

Beyond the key bitter compound kaempferol 3-*O*-(2‴-*O*-sinapoyl-β-d-sophoroside) previously described in the literature (**1**), eight further bitter and astringent-tasting kaempferol glucosides (**2**–**9**) have been identified in rapeseed protein isolates (*Brassica napus* L.). The bitterness and astringency of these taste-active substances have been described with taste threshold concentrations ranging from 3.3 to 531.7 and 0.3 to 66.4 μmol/L, respectively, as determined by human sensory experiments. In this study, the impact of **1** and kaempferol 3-*O*-β-d-glucopyranoside (**8**) on TAS2R-linked proton secretion by HGT-1 cells was analyzed by quantification of the intracellular proton index. mRNA levels of bitter receptors TAS2R3, 4, 5, 13, 30, 31, 39, 40, 43, 45, 46, 50 and TAS2R8 were increased after treatment with compounds **1** and **8**. Using quantitative UHPLC-MS/MS_MRM_ measurements, the concentrations of **1**–**9** were determined in rapeseed/canola seeds and their corresponding protein isolates. Depending on the sample material, compounds **1**, **3**, and **5**–**9** exceeded dose over threshold (DoT) factors above one for both bitterness and astringency in selected protein isolates. In addition, an increase in the key bitter compound **1** during industrial protein production (apart from enrichment) was observed, allowing the identification of the potential precursor of **1** to be kaempferol 3-*O*-(2‴-*O*-sinapoyl-β-d-sophoroside)-7-*O*-β-d-glucopyranoside (**3**). These results may contribute to the production of less bitter and astringent rapeseed protein isolates through the optimization of breeding and postharvest downstream processing.

## Introduction

Ten years ago, the Food and Agriculture Organization of the United Nations predicted that global protein demand will more than double by 2050 due to expected population growth.^1^ As increased consumption of animal-based proteins would have a negative environmental impact—both requiring more land and water than is sustainable and generating greenhouse gas emissions—the development of additional and more sustainable plant-based protein sources for direct human consumption is becoming more and more important.^2,3^ In addition to plant-based proteins from, for example, pumpkin seed, hemp seed, sunflower seed, potato, grains, and legumes (soy, peas, lentils, lupins, fava beans, mung beans, or chickpeas), rapeseed proteins are considered suitable supplements to the current protein supply.^3^ Rapeseed (*Brassica napus* L.) cultivars with reduced levels of erucic acid and glucosinolates, also called canola, are not only the second most important oil crop after soybeans in the world but also exhibit the potential to become one of the most productive domestic protein crops.^4^ Rapeseed press cake gained as a side stream during rapeseed oil production presents an excellent amino acid composition and nutritional value, but it has not yet been harnessed for broader utilization in human nutrition^5,6^ because of its intense, long-lasting bitter and astringent off-notes.^7−9^

To determine which nonvolatile key taste compounds are responsible for this long-lasting bitter aftertaste, we recently applied taste dilution analysis to a fraction prepared from a rapeseed protein isolate. This sensory-directed fractionation, together with a test reconstitution experiment, led to the identification of kaempferol 3-*O*-(2‴-*O*-sinapoyl-sophoroside; K3OSS, **1**) as the key player imparting bitterness to the analyzed rapeseed protein isolate.^10^

Over the course of the past decade, several studies have highlighted that a wide variety of nonvolatile secondary metabolites are sticky and noncovalently bind to proteins, causing bitter and astringent off-flavor impressions of plant-based protein isolates such as those produced from peas. Their concentrations are enriched during the production of the isolates, and they depend on raw material selection and isolate production.^10−12^ In addition to bitter stimuli **1**, several other kaempferol glycosides have been reported in rapeseed seeds and proteins without impacting their taste quality,^13−17^ which might be concentrated in various rapeseed protein products depending on their production and technological processing. The literature has indicated that depending on the positions and linkages of its glycosylations, kaempferol glycosides may exhibit a bitter and/or astringent flavor.^10,18,19^

To understand the degree to which kaempferol glycosides cause bitter and astringent off-taste impressions in rapeseed and rapeseed protein products, a rapeseed protein isolate was screened by means of UHPLC-ToF-MS to facilitate the isolation of those target compounds (**1**–**9**), elucidate their structure by applying NMR and MS experiments, and determine their human taste threshold. We also identified the bitter taste receptors mediating the bitter off-taste of kaempferol glycosides **1** and **8**. Furthermore, quantitative studies were performed to investigate the metabolomic changes of kaempferol glycosides during protein isolate processing.

## Materials and Methods

### Chemicals

Acetonitrile and methanol (J.T. Baker, Deventer, The Netherlands), formic acid (Merck, Darmstadt, Germany), d-galactose, l-glucose, d-glucose, d-glucuronic acid, d-galacturonic acid, l-rhamnose, l-tyrosine, phenylethyl-isothiocyanate, pyridine anhydrous, deuterated methanol, deuterated acetonitrile, deuterium oxide (Sigma-Aldrich, Steinheim, Germany), and rutin (Roth, Karlsruhe, Germany) were obtained commercially, and **1** was purified as reported recently.^10^ For UHPLC-MS/MS analysis, the acetonitrile was liquid chromatography–mass spectrometry (LC–MS) grade (Honeywell, Seelze, Germany). Purified water used for chromatography was obtained by an Advantage A 10 water system (Millipore, Molsheim, France). Bottled water (Evian, low mineralization: 405 mg/L) was adjusted to pH 5.9 with formic acid for sensory analysis. The cruciferin-rich and napin-rich proteins were produced by Pilot Pflanzenöltechnologie Magdeburg e.V. (PPM, Magdeburg, Germany) from the rapeseed variety Mentor obtained from Norddeutsche Pflanzenzucht Hans-Georg Lembke KG (NPZ, Holtsee, Germany). For the processing trial, between 7 and 10 members of 12 SSD families and 6 DH families were selected from a BnNAM (*B. napus* nested association mapping) population, grown and harvested at NPZ.^20,21^ Protein fractions of the resulting 150 samples were processed at PPM.^2121a^ Cell culture materials such as trypsin, penicillin/streptomycin, and fetal bovine serum were obtained by Pan-Biotech (Aidenbach, Germany). Dulbecco’s Modified Eagle Medium (DMEM) and nigericin were purchased from Thermo Fisher (Waltham, Massachusetts). Phosphate-buffered saline was obtained by Lonza Bio Whittaker (Basel, Switzerland), and histamine and kaempferol 3-*O*-β-d-glucoside were purchased from Sigma-Aldrich (St. Louis, Missouri). Cell viability was tested by means of 3-(4,5-dimethylthiazol-2-yl)-2,5-diphenyltetrazolium bromide (MTT, Carl Roth GmbH & Co. KG, Karlsruhe, Germany). For the detection of proton secretory activity, the fluorescent dye 1,5 -carboxy-seminaphtorhodafluor acetoxymethylester (SNARF-1-AM) was obtained from Thermo Fisher Scientific. For RNA isolation, we used the peqGold Micro Spin Total RNA Kit obtained by VWR (Radnor, Pennsylvania). The iScript gDNA Clear cDNA Synthesis Kit, the Sso Advanced universal SYBR Green Supermix, and the TAS2R primer were purchased from BioRad (Hercules, California).

### Solvent Extraction

According to Hald et al.,^10^ rapeseed protein isolate (300 g) was extracted 3 times with methanol/water (50/50, v/v; 1.5 L) by stirring for 30 min. After centrifugation (5 min, 5000 rpm) and filtration, the filtrates were combined and freed from the solvent in vacuum at 40 °C. After lyophilization, the methanol/water extractables (fraction I) were obtained and kept at −20 °C until further fractionation.

### Solid-Phase Extraction (SPE) of the Methanol/Water Extract

Solid-phase extraction was performed following Hald et al.^10^ A Chromabond C18ec polypropylene cartridge (Macherey-Nagel, Düren, Germany) was preconditioned with methanol (70 mL) followed by water (70 mL). Then, an aliquot (1 g) of fraction I was suspended in water (50 mL), applied on the column, and stepwise-eluted with water (75 mL), methanol/water (30/70, v/v, 75 mL), and methanol/water (50/50, v/v, 75 mL) to give fractions I-A to I-C. Fractions I-A and I-B were discarded after UPLC-ToF-MS screening, while fraction I-C was freed from the solvent via vacuum evaporation and lyophilization and stored at −20 °C until used for UPLC-ToF-MS screening, sensory analysis, or further fractionation.

### HPLC Fractionation of SPE Fraction I-C

Following Hald et al.,^10^ fraction C was dissolved in acetonitrile/water (20/80, v/v; 5 mg/mL) and, after membrane filtration, injected onto a 250 mm × 21 mm, 5 μm, Nucleodur C18 column (Macherey-Nagel, Düren, Germany). The separation was performed with a flow rate of 20 mL/min and 0.1% formic acid in water (solvent A) and acetonitrile (solvent B), monitoring the effluent at 220 nm and collecting the effluent into 18 subfractions using the following gradient: 0% B for 3 min, in 6 min to 20% B, keep 20% B for 3 min, in 6 min to 30% B, keep 30% B for 8 min, in 4 min to 100% B, keep 100% B for 3 min, in 5 min to 0% B, and keep it for 4 min at 0% B. The HPLC fractions of multiple runs were combined, freed from the solvent in vacuum (40 °C), lyophilized, and then used for LC-MS and NMR analysis.

### UHPLC-ToF-MS Analysis of HPLC Fractions I-C-1 to I-C-18

Aliquots (0.5 mg) of HPLC fractions I-C-1 to I-C-18 were dissolved in acetonitrile/water (20/80, v/v, 1 mL) and injected into an Acquity ultraperformance liquid chromatography (UPLC) core system connected to a Synapt G2-S HDMS spectrometer (Waters, Manchester, U.K.). The fractions were chromatographically separated on a 2.1 mm × 150 mm, 1.7 μm, BEH C18 column (Waters) operated at 45 °C using a flow rate of 0.4 mL/min, 0.1% aqueous formic acid (solvent A), and 0.1% formic acid in acetonitrile (solvent B), applying the following gradient: 0 min 5% B and 4 min 100% B.

ToF-MS analyses were performed in the negative electrospray ionization mode (ESI−) by using the following ion source parameters: capillary voltage: −2.5 kV, sampling cone: 50 V, source offset: 30 V, source temperature: 150 °C, desolvation temperature: 450 °C, cone gas: 2 L/h, nebulizer gasflow: 6.5 bar, and desolvation gas: 850 L/h. Data acquisition was accomplished with MassLynx 4.1 (Waters, Manchester, U.K.).

#### Isolation of Kaempferol 3-*O*-β-d-Sophoroside-7-*O*-β-d-glucopyranoside (**2**) from HPLC Fraction I-C-1

**Figure 1:**
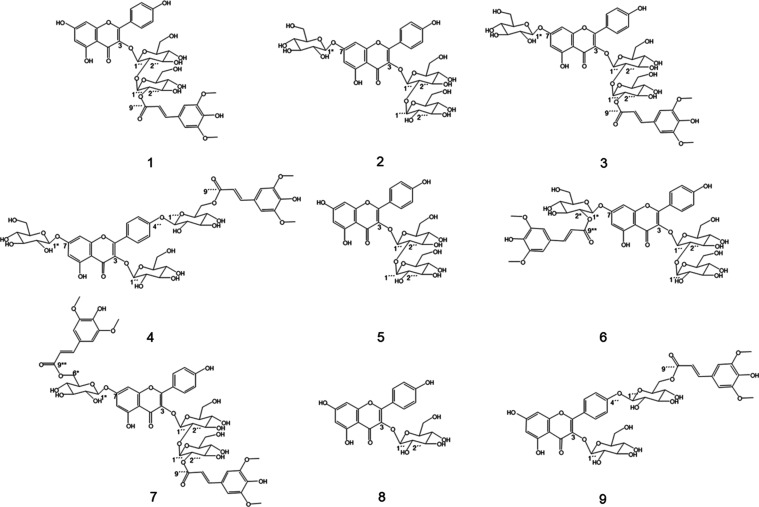
Chemical structures of the identified bitter and astringent-tasting compounds (no.) from rapeseed protein: kaempferol 3-*O*-(2‴-*O*-sinapoyl*-*β-d-sophoroside) (1), kaempferol 3-*O-*β-d-sophoroside-7-*O-*β-d-glucopyranoside (2), kaempferol 3-*O*-(2‴-*O*-sinapoyl*-*β-d-sophoroside)-7-*O-*β-d-glucopyranoside (3), kaempferol 4′-*O*-(6-*O*-sinapoyl*-*β-d-glucopyranoside)-3,7-di-*O-*β-d-glucopyranoside (4), kaempferol 3-*O-*β-d-sophoroside (5), kaempferol 3-*O-*β-d-sophoroside-7-*O*-(2-*O*-sinapoyl*-*β-d-glucopyranoside) (6), kaempferol 3-*O*-(2‴-*O*-sinapoyl*-*β-d-sophoroside)-7-*O*-(6-*O*-sinapoyl*-*β-d-glucopyranoside) (7), kaempferol 3-*O-*β-d-glucopyranoside (8), and kaempferol 4′-*O*-(6-*O*-sinapoyl*-*β-d-glucopyranoside)-3-*O-*β-d-glucopyranoside (9).

Unless otherwise specified, the fractions were dissolved in acetonitrile/water (1 mg/mL, 20/80, v/v), and after membrane filtration, kaempferols were purified with a HPLC (Jasco, Groß-Umstadt, Germany) consisting of two PU-2087 pumps, a UV-2075 ultraviolet (UV) Detector, and a Rh 7725 type Rheodyne injection valve (Rheodyne, Bensheim, Germany) via a semipreparative C18ec column (250 mm × 10 mm, 5 μm, 100 Å, Macherey-Nagel, Düren, Germany) with an operating flow rate of 4.7 mL/min and a detection wavelength of 220 nm. Chromatography was achieved by applying the following gradient: start at 0% B, in 3 min to 5% B; in 21 min to 18% B; in 5 min to 100% B; keep 100% B for 2 min, in 2 min to 0% B; keep 0% B for 2 min. The collected fractions were freed from the solvent in vacuum at 40 °C and freeze-dried twice. The obtained residue was then used for structural as well as taste threshold analysis. Based on MS/MS, ToF-MS, and one-dimensional (1D) and two-dimensional (2D) NMR spectroscopy results, the structure of the taste-active compound could be determined as kaempferol 3-*O*-β-d-sophoroside-7-*O*-β-d-glucopyranoside, **2** (Figure 1): LC-MS (ESI^–^): *m*/*z* 771.2 [M – H]^−^; LC-MS/MS (DP = −215 V): *m*/*z* 771 [M – H]^−^ (38%), 609 [M-H-Glc]^−^ (100%), 446 [M-H-Glc-Glc]^−^ (5%), 429 [M-H-Glc-Glc-H_2_O]^−^ (3%), 284 [M-H-Glc-Glc-Glc-H_2_O]^−^ (43%), 255 (51%); LC-ToF-MS: *m*/*z* 771.1990 (measured); *m*/*z* 771.1983 (calcd. for [C_33_H_39_O_21_]^−^); ^1^H NMR (500 MHz; CD_3_CN/D_2_O, 66/33, v/v): δ 8.05 [d, 2H *J* = 8.9 Hz, H–C(2′/6′)], 6.99 [d, 2H, *J* = 8.9 Hz, H–C(3′/5′)], 6.78 [d, 1H, *J*_6,8_ = 2.1 Hz, H–C(8)], 6.52 [d, 1H, *J*_6,8_ = 2.1 Hz, H–C(6)], 5.18 [d, 1H, *J*_1″,2″_ = 8.0 Hz, H–C(1″)], 5.15 [d, 1H, *J*_1*,2*_ = 8.0 Hz, H–C(1*)], 4.86 [d, 1H, *J*_1‴,2‴_ = 8.0 Hz, H–C(1‴)], 3.94 [dd, 1H, *J*_6*A,6*B_ = 12.3 Hz, *J*_6*A,5*_ = 1.9 Hz, H–C(6*A)], 3.88 [dd, 1H, *J*_1″,2″_ = 8.0 Hz, *J*_2″,3″_ = 9.8 Hz, H–C(2″)], 3.83 [d, 1H, *J*_6‴A,6‴B_ = 12.3 Hz, H–C(6‴A)], 3.77 [dd, 1H, *J*_6*B, 5*_ = 5.5 Hz, *J*_6*B,6*A″_ = 12.3 Hz, H–C(6*B)], 3.71–3.67 [m, 1H, H–C(6‴B)], 3.66–3.61 [m, 3H, H–C(6″A,3″,5‴)], 3.60–3.57 [m, 2H, H–C(5*,2*)], 3.55–3.54 [m, 1H, H–C(6″B)], 3.53–3.47 [m, 2H, H–C(3*,4*)], 3.46–3.42 [m, 1H, H–C(4″)], 3.40–3.38 [m, 2H, H–C(4‴,3‴)], 3.36–3.33 [dd, *J*_1‴,2‴_ = 8.0 Hz, *J*_2‴,3‴_ = 9.3 Hz, 1H, H–C(2‴)], 3.27–3.24 [dd, 1H, *J*_5″,4″_ = 10 Hz, *J*_5″,6″B_ = 5.3 Hz, *J*_5″,6″A_ = 2.1 Hz H–C(5″)]. ^13^C NMR (125 MHz; CD_3_CN/D_2_O, 66/33, v/v): δ 177.7 [C(4)], 161.6 [C(7)], 159.4 [C(5)], 158.3 [C(4′)], 157.8 [C(2)], 155.6 [C(8a)], 133.1 [C(3)], 130.6 [C(2′/6′)], 120.6 [C(1′)], 114.6 [C(3′/5′)], 105.7 [C(4a)], 101.6 [C(1‴)], 98.8 [C(1″)], 98.7 [C(1*)], 98.6 [C(6)], 94.5 [C(8)], 78.8 [C(2″)], 75.4 [C(3″)], 75,2 [C(5″/5‴,3‴)], 74.8 [C(3*)], 74.61 [C(5*)], 72.8 [C(2‴)], 71.8 [C(2*)], 68.7 [C(4‴)], 68.4 [C(4*)], 68.2 [C(4″)], 60.1 [C(6″)], 59.6 [C(6*)], 59.5 [C(6‴)].

#### Isolation of Kaempferol 3-*O*-(2‴-*O*-Sinapoyl-β-d-sophoroside)-7-*O*-β-d-glucopyranoside (**3**) from HPLC Fraction I-C-2

The following gradient with the detection wavelength of 220 nm was applied: start at 0 min and keep it for 3 min at 0%; in 21 min to 18% B; in 5 min to 100% B; keep it for 2 min at 100% B; in 2 min to 0% B and keep it for 2 min at 0% B.

##### Kaempferol 3-*O*-(2‴-*O*-Sinapoyl-β-d-sophoroside)-7-*O*-β-d-glucopyranoside, **3** (Figure 1)

LC-MS (ESI^–^): *m*/*z* 977.2 [M – H]^−^; LC-MS/MS (DP = −40 V): *m*/*z* 977 [M – H]^−^ (92%), 815 [M-H-Glc]^−^ (100%), 623 (5%), 609 [M-H-Glc-Sinapoyl]^−^ (33%), 591 [M-H-Glc-Sinapoyl-H2O]^−^ (14%), 446 [M-H-Glc-Glc-Sinapoyl]^−^ (8%), 429 [M-H-Glc-Glc-Sinapoyl-H_2_O]^−^ (4%), 284 [M-H-Glc-Glc-Glc-Sinapoyl-H_2_O]^−^ (62%), 255 (55%); LC-ToF-MS: *m*/*z* 977.2563 (measured); *m*/*z* 977.2663 (calcd. for [C_44_H_49_O_25_]^−^); ^1^H NMR (500 MHz; DMSO-*d*_6_): δ 7.99 [d, 2H, *J* = 8.9 Hz, H–C(2′/6′)], 7.42 [d, 1H, *J* = 15.8 Hz, H–C(7‴′)], 6.91 [d, 2H, *J* = 8.9 Hz, H–C(3′/5′)], 6.79 [s, 2H, H–C(2‴′/6‴′)], 6.69 [d, 1H, *J*_6,8_ = 2.1 Hz, H–C(8)], 6.40 [d, 1H, *J*_6,8_ = 2.1 Hz, H–C(6)], 6.38 [d, 1H, *J* = 15.8 Hz, H–C(8‴′)], 5.76 [d, 1H, *J*_1″,2″_ = 8 Hz, H–C(1″)], 5.09 [d, 1H, *J*_1‴,2‴_ = 8.0 Hz, H–C(1‴)], 5.06 [d, 1H, *J*_1*,2*_ = 8.0 Hz, H–C(1*)], 4.70 [dd, 1H, *J*_1″,2″_ = 8.0 Hz, H–C(2‴)], 3.73 [s, 1H, H–C(3′/5′-Ome)], 3.70 [m, 1H, H–C(6‴A)], 3.50–3.41 [m, 8H, H–C(6″A, 6*A,6‴B,5″,3‴,3*,2″,2‴)], 3.34–3.15 [m, 5H, H–C(5‴,4‴,4*,3″,2*)], 3.06 [m, 2H, H–C(5*,4″)]. ^13^C NMR (125 MHz; DMSO-*d*_6_): δ 177.7 [C(4)], 166.2 [C(9‴′)], 163.0 [C(7)], 160.9 [C(5/4′)], 157.8 [C(2)], 156.2 [C(8a)], 148.2 [C(3‴′/5‴′)], 145.3 [C(7‴′)], 138.5 [C(4‴′)], 133.4 [C(3)], 131.5 [C(2′/6′)], 124.7 [C(1‴′)], 120.9 [C(1′)], 115.8 [C(3′/5′)], 115.7 [C(8‴′)], 106.1 [C(2‴′/6‴′)], 105.9 [C(4a)], 100.2 [C(1*)], 99.6 [C(6)], 99.3 [C(1‴)], 97.3 [C(1″)], 94.7 [C(8)], 79.7 [C(2″)], 77.6 [C(5*)], 77.5 [C(5‴)], 77.2 [C(5″)], 76.6 [C(3″)], 76.3 [C(3*)], 74.7 [C(3‴)], 74.0 [C(2‴)], 73.4 [C(2*)], 70.4 [C(4″/4*)], 69.9 [C(4‴)], 61.2 [C(6″)], 60.9 [C(6*)], 60.8 [C(6‴)], 56.3 [C(3‴′/5‴′Ome)].

#### Isolation of Kaempferol 4′-(6-*O*-Sinapoyl-β-d-glucopyranoside)-3,7-di-*O*-β-d-glucopyranoside (**4**) from HPLC Fraction I-C-4

Chromatography was obtained at 220 nm by applying the following gradient: start at 0% B; in 3 min to 5% B; in 6 min to 15% B; in 13 min to 30% B; in 2 min to 100% B; keep 100% B for 2 min; in 2 min to 0% B and keep it at 0% B for 2 min.

##### Kaempferol 4′-(6-*O*-Sinapoyl-*O*-β-d-hexopyranoside)-3,7-di-*O*-β-d-hexopyranoside, **4** (Figure 1)

LC-MS (ESI^–^): *m*/*z* 977.2 [M-H]^−^; LC-MS/MS (DP = −205 V): *m*/*z* 977 [M – H]^−^ (100%), 815 [M-H-Glc]^−^ (93%), 623 (3%), 609 [M-H-Glc]^−^ (17%), 591 [M-H-Glc-Sinapoyl-H_2_O]^−^ (1%), 446 [M-H-Glc-Glc-Sinapoyl]^−^ (25%), 429 [M-H-Glc-Glc-Sinapoyl-H_2_O]^−^ (4%), 284 [M-H-Glc-Glc-Glc-Sinapoyl-H_2_O]^−^ (46%), 255 (44%); LC-ToF-MS: *m*/*z* 977.2563 (measured); *m*/*z* 978.2663 (calcd. for [C_44_H_49_O_25_]^−^); ^1^H NMR (500 MHz; DMSO-*d*_6_): δ 8.13 [d, 2H, *J* = 8.9 Hz, H–C(2′/6′)], 7.56 [d, 1H, *J* = 15.8 Hz, H–C(7‴′)], 7.19 [d, 2H, *J* = 8.9 Hz, H–C(3′/5′)], 7.01 [s, 2H, H–C(2‴′/6‴′)], 6.71 [d, 1H, *J*_6,8_ = 2.1 Hz, H–C(8)], 6.56 [d, 1H *J* = 15.8 Hz, H–C(8‴′)], 6.47 [d, 1H, *J*_6,8_ = 2.1 Hz, H–C(6)], 5.50 [d, 1H, *J*_1″,2″_ = 8.0 Hz, H–C(1″)], 5.15 [d, 1H, *J*_1‴,2‴_ = 8.0 Hz, H–C(1‴)], 5.09 [d, 1H, *J*_1*,2*_ = 7.6 Hz, H–C(1*)], 4.38 [m, 1H, H–C(6‴A)], 4.31 [ddd, 1H, H–C(6‴B)], 3.76–3.73 [m, 3H, H–C(3′/5′-Ome, 5‴)], 3.74–3.68 [m, 1H, H–C(6*A)], 3.59 [dd, 1H, *J*_6″A, 5″_ = 5.0 Hz, *J*_6″A,6″B_ = 11.3 Hz H–C(6″A)], 3.39–3.09 [m, 13H, H–C(6*B, 6″A, 5*,5″,4*,4‴,4″,3*,3‴,3″,2*,2‴,2″)]. ^13^C NMR (125 MHz; DMSO-*d*_6_): δ 178.2 [C(4)], 167.1 [C(9‴′)], 163.4 [C(7)], 161.3 [C(5)], 159,6 [C(4′)], 156.7 [C(2)], 156.5 [C(8a)], 148.4 [C(3‴′/5‴′)], 146.1 [C(7‴′)], 134.4 [C(3)], 131.2 [C(2′/6′)], 124.1 [C(1‴′/1′)], 116.2 [C(3′/5′)], 114.9 [C(8‴′)], 106.7 [C(2‴′/6‴′)], 106.2 [C(4a)], 101.1 [C(1″)], 100.2 [C(1‴)], 100.1 [C(1*)], 99.8 [C(6)], 94.8 [C(8)], 78.1 [C(5″)], 77.6 [C(5*)], 76.8 [C(3″/3*)], 76.7 [C(3‴)], 74.6 [C(2″)], 74.2 [C(5‴)], 73.6 [C(2‴)], 73.5 [C(2*)], 70.3 [C(4‴)], 70.0 [C(4″)], 69.8 [C(4*)], 63.4 [C(6‴)], 61.3 [C(6″)], 61.0 [C(6*)], 56.4 [C(3‴′/5‴′Ome)].

#### Identification of Kaempferol 3-*O*-β-d-Sophoroside (**5**) in HPLC Fraction I-C-6

For fraction I-C-6, no further fractionation was needed. The bitter tastant kaempferol 3-*O*-β-d-sophoroside was identified using MS/MS, ToF-MS, as well as 1D and 2D NMR experiments, **5** (Figure 1): LC-MS (ESI^–^): *m*/*z* 609.0 [M – H]^−^; LC-MS/MS (DP = −195 V): *m*/*z* 609 [M – H]^−^ (38%), 609 [M-H-Glc]^−^ (100%), 447 [M-H-Glc-Glc]^−^ (8%), 429 [M-H-Glc-Glc-H_2_O]^−^ (1%), 284 [M-H-Glc-Glc-Glc-H_2_O]^−^ (48%), 255 (38%); LC-ToF-MS: *m*/*z* 609.1451 (measured); *m*/*z* 609.1456 (calcd. for [C_27_H_29_O_16_]^−^); ^1^H NMR (400 MHz; CD_3_CN/D_2_O, 66/33, v/v): δ 8.00 [d, 2H, *J* = 8.9 Hz, H–C(2′/6′)], 6.93 [d, 2H, *J* = 8.9 Hz, H–C(3′/5′)], 6.45 [d, 1H, *J*_6,8_ = 2.1 Hz, H–C(8)], 6.24 [d, 1H, *J*_6,8_ = 2.1 Hz, H–C(6)], 5.19 [d, 1H, *J*_1″,2″_ = 7.6 Hz, H–C(1″)], 4.75 [d, 1H, *J*_1‴,2‴_ = 7,8 Hz, H–C(1‴)], 3.75–3.68 [m, 2H, H–C(2″/6‴A)], 3.59–3.24 [m, 9H, H–C(6‴A/6‴B/6″A/5″/4‴/4″/3‴/3″/2‴)], 3.15 [m, 1H, H–C(5‴)]. ^13^C NMR (100 MHz; CD_3_CN/D_2_O, 66/33,v/v): δ 178.2 [C(4)], 163.8 [C(7)], 160.0 [C(5)], 159.4 [C(4′)], 157.8 [C(2)], 156.8 [C(8a)], 133.5 [C(3)], 131.2 [C(2′/6′)], 121.4 [C(1′)], 115.3 [C(3′/5′)], 104.6 [C(4a)], 102.6 [C(1‴)], 99.4 [C(1″)], 98.8 [C(6)], 94.1 [C(8)], 80.0 [C(2″)], 76.1 [C(5″)], 76.0 [C(5‴,3″)], 75.8 [C(3‴)], 73.7 [C(2‴)], 69.5 [C(4‴)], 68.9 [C(4″)], 60.9 [C(6‴)], 60.4 [C(6″)].

#### Isolation of Kaempferol 3-*O*-Sophoroside-7-*O*-(2*-*O*-sinapoyl-β-d-glucopyranoside) (**6**) from HPLC Fraction I-C-7

In fraction I-C-7, the bitter tastant could be determined using MS/MS, ToF-MS, and 1D and 2D NMR experiments as kaempferol 3-*O*-sophoroside-7-*O*-(2*-*O*-sinapoyl-β-glucopyranoside), **6** (Figure 1): LC-MS (ESI^–^): *m*/*z* 977.2 [M – H]^−^; LC-MS/MS (DP = −25 V): *m*/*z* 977 [M – H]^−^ (100%), 815 [M – H]^−^ (3%), 609 [M-H-Sinapoyl]^−^ (67%), 429 [M-H-Sinapoyl-H_2_O-Glc]^−^ (4%), 284 [M-H-Sinapoyl-2Glc]^−^ (66%), 255 (50%); LC-ToF-MS: *m*/*z* 977.2571 (measured); *m*/*z* 978.2663 (calcd. for [C_44_H_49_O_25_]^−^); ^1^H NMR (400 MHz; CD_3_CN/D_2_O, 66/33, v/v): δ 7.95 [d,2H. *J* = 8.9 Hz, H–C(2′/6′)], 7.65 [d, 1H, *J*_7**,8**_ = 15.6 Hz, H–C(7**)], 6.92 [d, 2H, *J* = 8.9 Hz, H–C(3′/5′)], 6.83 [s, 2H, H–C(2**/6**)], 6.52 [d, 1H, *J*_6,8_ = 2.1, H–C(8)], 6.41 [d, 1H, *J* = 15.6 Hz, H–C(8**)], 6.30 [d, 1H, *J*_6,8_ = 2.1 Hz, H–C(6)], 6.14 [d, 1H, *J*_1*,2*_ = 8.2 Hz, H–C(1*)], 6.10 [d, 1H, *J*_1″,2″_ = 7.8 Hz, H–C(1″)], 5.00 [dd, 1H, *J*_1*,2*_ = 8.2 Hz, J_2*,3*_ = 9.5 Hz H–C(2*)], 4.74 [d, 1H, *J*_1‴,2‴_ = 7.8 Hz, H–C(1‴)], 3.85 [dd, 1H, *J*_6*B,5*_ = 1.9 Hz, *J*_6*B,6*A″_ = 12.3 Hz H–C(6*B)], 3.75–3.23 [m, 15H, H–C(6*B/6‴A/6‴B/6″A/6″B/5*/5″/4*/4‴/4″/3*/3‴/3″/2‴/2″)], 3.73 [s, 2H, H–C(3**/5**Ome)], 3.12 [m, 1H, H–C(5″)]. ^13^C NMR (100 MHz; CD_3_CN/D_2_O, 66/33, v/v): δ 178.2 [C(4)], 166.8 [C(9**)], 162.1 [C(7)], 160.8 [C(5)], 159.5 [C(4′)], 158.2 [C(2)], 156.1 [C(8a)], 147.6 [C(3**/5**)], 146.6 [C(7**)], 137.7 [C(4**)], 133.8 [C(3)], 131.3 [C(2′/6′)], 125.1 [C(1**)], 121.1 [C(1′)], 115.3 [C(3′/5′)], 114.3 [C(8**)], 106.3 [C(4a)], 105.8 [C(2**/6**)], 102.5 [C(1‴)], 99.5 [C(1″)], 99.0 [C(6)], 98.1 [C(1*)], 95.2 [C(8)], 79.9 [C(2″)], 76.5 [C(5*)], 76.0 [C(5‴)], 76.0 [C(5″/3″)], 75.7 [C(3*)], 73.7 [C(3‴/2‴)], 73.1 [C(2*)], 69.5 [C(4″)], 69.3 [C(4*)], 68.8 [C(4‴)], 60.9 [C(6″)], 60.5 [C(6*)], 60.3 [C(6‴)], 55.9 [C(3**/5**Ome)].

##### Isolation of Kaempferol 3-*O*-(2‴-*O*-Sinapoyl-β-d-sophoroside)-7-*O*-(6-*O*-sinapoyl-β-d-glucopyranoside) (**7**) from HPLC fraction I-C-9

By applying the following gradient: Start at 0% B; in 3 min to 10% B; in 7 min to 25% B; in 6 min to 28% B; in 5 min to 100% B; keep 100% B for 2 min; in 2 min to 0% B; keep 0% B for 2 min, the effluent was detected at 220 nm.

##### Kaempferol 3-*O*-(2‴-*O*-Sinapoyl-β-d-sophoroside)-7-*O*-(6-*O*-sinapoyl-β-d-glucopyranoside), **7** (Figure 1)

LC-MS (ESI^–^): *m*/*z* 1183.4 [M – H]^−^; LC-MS/MS (DP = −15 V): *m*/*z* 1183 [M – H]^−^ (100%), 977 [M-H-Sinapoyl]^−^ (2%), 815 [M-H-Sinapoyl-Glc]^−^ (62%), 609 [M-H-Sinapoyl-Sinapoyl-Glc]^−^ (18%), 429 [M-H-Glc-Glc-Sinapoyl-Sinapoyl-H_2_O]^−^ (3%), 284 [M-H-Glc-Glc-Glc-Sinapoyl-Sinapoyl-H_2_O]^−^ (23%), 254 (16%); LC-ToF-MS: *m*/*z* 1183.3147 (measured); *m*/*z* 1183.3142 (calcd. for [C_55_H_59_O_29_]^−^); ^1^H NMR (500 MHz; MeOD-*d*_3_): δ 7.85 [d, 2H, *J* = 8.9 Hz, H–C(2′/6′)], 7.63 [d, 1H, *J* = 15.8 Hz, H–C(7**)], 7.28 [d, 1H *J*_7‴′,8‴′_ = 15.8 Hz, H–C(7‴′)], 6.83 [d, 2H, *J* = 8.9 Hz, H–C(3′/5′)], 6.78 [s, 2H, H–C(2**/6**)], 6.50 [d, 1H, *J*_6,8_ = 2.1 Hz, H–C(6)], 6.42 [d, 1H, *J*_6,8_ = 8.8 Hz, H–C(8**)], 6.39 [d, 1H, *J*_6,8_ = 2.1 Hz, H–C(8)], 6.23 [s, 2H, H–C(2‴′/6‴′)], 6.11 [d, 1H, *J*_1″,2″_ = 8.1 Hz, H–C(1″)], 6.05 [d, 1H, *J*_7‴′,8‴′_ = 15.8 Hz, H–C(8‴′)], 5.21 [d, 1H, *J*_1‴,2‴_ = 8.0 Hz, H–C(1‴)], 5.11 [d, 1H, *J*_1*,2*_ = 7.4 Hz, H–C(1*)], 4.94 [dd, 1H, J_1‴,2‴_ = 8.0 Hz, *J*_2‴,3‴‴_ = 9.6 Hz, H–C(2‴)], 4.69 [dd, 1H, *J*_6*A,6*B_ = 12.1 Hz, *J*_6*A,5*_ = 2.1 Hz, H–C(6*A)], 4.35 [dd, 1H, *J*_6*A,6*B_ = 12.1 Hz, *J*_6*B,5*_ = 7.0 Hz, H–C(6*A)], 3.95–3.40 [m, 13H, H–C(6″A, 6‴A, 6″B, 6‴B, 5‴,5*,4‴,4*,3″,3‴,3*,2″,2*)], 3.28 [m, 2H, H–C(5″,4″)]. ^13^C NMR (125 MHz; MeOD-*d*_3_): δ 179.2 [C(4)], 168.9 [C(9**)], 168.4 [C(9‴′)], 164.3 [C(7)], 162.5 [C(5)], 161.5 [C(4′)], 157.7 [C(2)], 157.3 [C(8a)], 149.3 [C(3**/5**)], 148.8 [C(3‴′/5‴′)], 147.6 [C(7**)], 146.4 [C(7‴′)], 139.6 [C(4**)], 138.8 [C(4‴′)], 134.8 [C(3)], 132.1 [C(2′/6′)], 126.4 [C(1**)], 126.1 [C(1‴′)], 122.8 [C(1′)], 116.1 [C(3′/5′)], 116.0 [C(8‴′)], 115.7 [C(8**)], 107.5 [C(4a)], 106.8 [C(2**/6**)], 105.7 [C(2‴′/6‴′)], 101.7 [C(1*)], 100.5 [C(6)], 98.6 [C(1‴)], 97.2 [C(1″)], 95.6 [C(8)], 82.0 [C(2″)], 78.5 [C(5″)], 77.9, 77.6 [C(5″,5*)], 75.9 [C(3‴)], 75.6 [C(3″)], 75.3 [C(2‴)], 75.0 [C(3*)], 74.7 [C(2*)], 71.8 [C(4*)], 71.5 [C(4″)], 71.2 [C(4‴)], 64.7 [C(6*)], 62.3, 62.6 [C(6‴,6‴)], 56.8 [C(3**/5**Ome)], 56.3 [C(3‴′/5‴′Ome)].

#### Isolation of Kaempferol 3-*O*-β-d-Glucopyranoside (**8**) from HPLC Fraction I-C-11

The following gradient was applied to achieve the separation: start at 0% B; keep 0% B for 2 min; in 3 min to 25% B; in 13 min to 28% B; in 2 min to 100% B; keep 100% B for 2 min; in 3 min to 0% B; keep 0% B for 2 min.

##### Kaempferol 3-*O*-β-d-Glucopyranoside, **8** (Figure 1)

LC-MS (ESI^–^): *m*/*z* 447.2 [M – H]^−^; LC-MS/MS (DP = −95 V): *m*/*z* 447 [M – H]^−^ (100%), 284 [M – H]^−^ (45%), 254 (55%); LC-ToF-MS: *m*/*z* 447.0938 (measured); *m*/*z* 447.0927 (calcd. for [C_21_H_19_O_11_]^−^); ^1^H NMR (400 MHz; CD_3_CN/D_2_O, 66/33, v/v): δ 8.01 [d, 2H, *J* = 8.0 Hz, H–C(2′/6′)], 6.92 [d, 2H, *J* = 8.0 Hz, H–C(3′/5′)], 6.45 [s, 1H, H–C(8)], 6.24 [1H, H–C(6)], 5.00 [s, 1H, H–C(1″)], 3.51–3.27 [m, 6H, H–C(6″A/6″B/5″/4″/3″/2″)], 3.12 [m1H, H–C(5″)]. ^13^C NMR (100 MHz; CD_3_CN/D_2_O, 66/33, v/v): δ 178.2 [C(4)], 164.3 [C(7)], 161.0 [C(5)], 159.6 [C(4′)], 158.0 [C(2)], 157.1 [C(8a)], 134.2 [C(3)], 131.2 [C(2′/6′)], 121.5 [C(1′)], 115.2 [C(3′/5′)], 104.5 [C(4a)], 102.7 [C(1″)], 99.0 [C(6)], 94.2 [C(8)], 76.2 [C(5″)], 75.9 [C(3″)], 73.8 [C(2″)], 69.2 [C(4″)], 60.7 [C(6″)].

#### Isolation of Kaempferol 4′-(6-*O*-Sinapoyl-β-d-glucopyranoside)-3-*O*-β-d-glucopyranoside (**9**) from HPLC Fraction I-C-12

Fraction I-C-12 was purified using the same gradient applied for I-C-11 and led to the identification of kaempferol 4′-(6-*O*-sinapoyl-β-d-glucopyranoside)-3-*O*-β-d-glucopyranoside, **9** (Figure 1): LC-MS (ESI^–^): *m*/*z* 977.2 [M – H]^−^; LC-MS/MS (DP = −30 V): *m*/*z* 815 [M – H]^−^ (100%), 653 (37%), 447 [M-H-Glc-Sinapoyl]^−^ (19%), 285 [M-H-Glc-Glc-Sinapoyl-H_2_O]^−^ (56%), 255 (28%); LC-ToF-MS: *m*/*z* 815.2040 (measured); *m*/*z* 815.2034 (calcd. for [C_38_H_39_O_20_]^−^); ^1^H NMR (500 MHz; DMSO-*d*_6_): δ 8.04 [d, 2H, *J* = 8.9 Hz, H–C(2′/6′)], 7.52 [d, 1H, *J*_7‴′,8‴′_ = 15.8 Hz, H–C(7‴′)], 7.14 [d, 2H, *J* = 8.9 Hz, H–C(3′/5′)], 6.92 [s, 2H, H–C(2‴′/6‴′)], 6.48 [d, 1H, *J*_7‴′/8‴′_ = 15.8 Hz, H–C(8‴′)], 6.33 [d, 1H, *J*_6,8_ = 2.1 Hz, H–C(8)], 6.20 [d, 1H, *J*_6,8_ = 2.1 Hz, H–C(6)], 5.38 [d, 1H, *J*_1″,2″_ = 7.4 Hz, H–C(1″)], 5.07 [d, 1H, *J*_1‴,2‴_ = 8 Hz, H–C(1‴)], 4.38 [d, 1H, *J*_6‴A,6‴B_ = 11.8 Hz, H–C(6‴A)], 4.31 [dd, 1H, J_6‴A,6‴B_ = 11.8 Hz, J_6‴B,5‴_ = 5.5 Hz, H–C(6‴B)], 3.78–3.73 [m, 1H, H–C(5‴)], 3.70 [s, 2H, H–C(3′/5′-Ome)], 3.55 [d, 1H, *J*_6″A,6″B_ = 11.8 Hz, H–C(6″A)], 3.39–3.30 [m, 4H, H–C(6″B,4‴,3‴,2‴)], 3.23–3.08 [m, 4H, H–C(5″,4″,3″,2″)]. ^13^C NMR (125 MHz; DMSO-*d*_6_): δ 177.9 [C(4)], 167.3 [C(9‴′)], 164.9 [C(7)], 161.4 [C(5)], 159,4 [C(4′)], 157.6 [C(2)], 156.3 [C(8a)], 148.4 [C(3‴′/5‴′)], 146.2 [C(7‴′)], 134.2 [C(3)], 131.2 [C(2′/6′)], 124.8 [C(1‴′)], 124.3 [C(1′)], 116.3 [C(3′/5′)], 115.1[C(8‴′)], 106.4 [C(2‴′/6‴′)], 104.4 [C(4a)], 101.4 [C(1″)], 100.1 [C(1‴)], 99.3 [C(6)], 94.3 [C(8)], 77.8 [C(5″)], 76.6 [C(3″)], 76.4 [C(3‴)], 74.3 [C(2″)], 74.1 [C(5‴)], 73.4 [C(2‴)], 70.1 [C(4‴)], 69.9 [C(4″)], 63.5 [C(6‴)], 61.1 [C(6″)], 56.5 [C(3‴′/5‴′)].

### Acid Hydrolysis of Kaempferols for the Determination of Monosaccharides

To determine the carbohydrate moieties attached to kaempferol derivatives, compounds **1**–**9** were hydrolyzed and analyzed according to the literature.^22,23^ For acidic hydrolysis, HCl (1 N, 200 μL) was added to an aliquot of each compound (60 μL) and heated for 1 h at 100 °C. The mixtures were evaporated to dryness under reduced pressure, the residues were dissolved in H_2_O (750 μL), and then extracted with EtOAc (2 μL × 750 μL). To obtain the monosaccharides, the H_2_O layers were dried under nitrogen flow. To each residue, l-cystein methyl ester hydrochloride dissolved in anhydrous pyridine (2 mg/mL) was added. Each solution was equilibrated for 1 h at 60 °C (1400 rpm). Afterward, phenylethyl-isothiocyanate (5 μL) was added. The solution was shaken for 1 h at 60 °C with 1400 rpm. The mixture was dried under a nitrogen stream and the resulting residues were resolved in CH_3_CN/H_2_O (500 μL, 1/1, v/v). Aliquots (0.5 μL) of each solution were analyzed by means of UHPLC-MS/MS using a Kinetex F5 column (100 mm × 2.1 mm i.d., 100 Å, 1.7 μm, Phenomenex, Aschaffenburg, Germany) with a flow rate of 0.4 mL/min for chromatographic separation and the mobile phase consisted of (A) formic acid (1% in H_2_O) and (B) CH_3_CN (with 1% formic acid) using the following gradient: 0 min, 5% B; 3 min, 5% B; 5 min, 20% B; 25 min, 25% B; 27 min, 100% B; 30 min, 100% B; 31 min, 5% B; 35 min, 5% B. The reference compounds d-galactose, l-glucose, d-glucose, d-glucuronic acid, d-galacturonic acid, and l-rhamnose were prepared similarly. The following MRM transitions were used to analyze the derivatized monosaccharides: Q1/Q3 of *m*/*z* 461.0/298.1 (DP = 86 V, CE = 17 V, CXP = 6 V) for d-glucose, Q1/Q3 of *m*/*z* 461.0/298.1 (DP = 71 V, CE = 17 V, CXP = 6 V) for l-glucose, Q1/Q3 of *m*/*z* 461.1/298.2 (DP = 71 V, CE = 17 V, CXP = 6 V) for d-galactose, Q1/Q3 of *m*/*z* 475.0/312.1 (DP = 91 V, CE = 19 V, CXP = 6 V) for d-galacturonic acid, Q1/Q3 of *m*/*z* 475.0/312.1 (DP = 61 V, CE = 19 V, CXP = 8 V)for d-glucuronic acid, and Q1/Q3 of *m*/*z* 445.0/282.1 (DP = 61 V, CE = 17 V, CXP = 6 V) for l-rhamnose. The following retention times were observed: d-galactose,11.4 min; l-glucose, 12.0 min; d-glucose, 12.2 min; d-glucuronic acid, 12.4 min; d-galacturonic acid, 12.6; and l-rhamnose, 16.6 min.

### Sensory Analysis

#### Sensory Panel Training and Sample Pretreatment

The sensory panel consisted of 22 panelists (11 females, 11 males, 23–33 years of age) who underwent weekly training sessions after they had given informed consent to participate in the sensory tests.^19,24,25^ To avoid sensory cross-model interactions with odorants, the sensory test was performed while wearing a nose clip. The analyses were performed at 22–25 °C in a sensory panel room.

#### Human Taste Recognition Thresholds

The threshold concentration at which the bitter and astringent taste quality of compounds **1**–**9** was just detectable was determined with a two-alternative forced choice test (2-AFC). The purified substances were solved in bottled water with ascending levels in concentration. The average human taste threshold values for the bitter and astringent taste of compounds **1**–**9** are summarized in Table 1.

**Table 1:** Human Taste Recognition Thresholds for Compounds **1**–**9**

compound no.	bitter threshold conc. [μmol/L]	astringent threshold conc. [μmol/L]
**1**	3.4^a^	0.3
**2**	184.0	4.8
**3**	160.7	19.4
**4**	320.8	11.5
**5**	531.7	66.4
**6**	243.9	35.1
**7**	265.1	16.6
**8**	324.7	29.7
**9**	149.3	8.1

a Bitter taste threshold taken from Hald et al.^10^

### Quantitation of **1**–**9**

#### Sample Workup

Rapeseed protein or rapeseed seeds (200 mg) were weighted in a bead beater tube (CK28_15 mL, Bertin Technologies, Montigny-le-Bretonneux, France) filled with ceramic balls (2.8 mm). Methanol/water (80/20, v/v; 5 mL) and internal standard (IS) solution (10 μL, rutin, 52.25 mg/L) were added to the tube, and the sample was extracted (3 s × 30 s with 20 s breaks, 6000 rpm) with a bead beater (Precellys Homogenizer, Bertin Technologies). The extract was equilibrated on a shaking plate (GFL-3005 Orbital Shaker, GFL, Germany) for 30 min and then centrifuged (5 min, 4000 rpm) using an Eppendorf Centrifuge 5702 (Eppendorf, Germany). The supernatant was membrane-filtered and analyzed by means of LC-MS/MS.

#### Calibration Curve and Linear Range

To quantify compounds **1**–**9**, a stock solution (54.6 mg/L) of **1** was prepared in acetonitrile/water (20/80, v/v). The stock solution was diluted to 1:2; 1:4; 1:10; 1:20; 1:40; 1:100; 1:200; 1:400, and 1:1000 with acetonitrile/water (20/80; v/v). An aliquot (100 μL) of each dilution step and the stock solution were spiked with 10 μL of the IS and then analyzed by means of LC-MS/MS using the characteristic MRM transitions (Table 2). These led to the following calibration curve: *y* = 403.72*x* – 0.0066 (*R*^2^ = 0.9972). The calibration curve was used for the quantification of all kaempferol glycosides.

**Table 2:** MRM Transitions and Optimized Parameters of Compounds **1**–**9** and the Internal Standard (**IS)^a^**

compound no.	Q1 (*m/z*)	Q3 (*m/z*)	DP (V)	CE (V)	CXP (V)
**1**	815.2	283.4	–150	–58	–9
**2**	771.1	609.1	–215	–38	–9
**3**	977.1	815.1	–40	–46	–5
**4**	977.1	815.2	–205	–44	–5
**5**	608.9	284.0	–195	–48	–13
**6**	977.1	609.1	–25	–52	–9
**7**	1183.1	815.1	–15	–52	–5
**8**	446.9	283.9	–95	–38	–5
**9**	815.1	284.5	–30	–60	–9
**IS**	609.1	299.9	–170	–50	–9

a Q1, Scant m/z in Quadrupole 1; Q3, scant m/z in quadrupole 3; DP, declustering potential; CE, collision energy; CXP, collision exit potential.

#### Recovery

The recovery was determined by spiking 100 μL of two different concentrations of analytes **1**–**9** (spiking experiment 1: **1** (0.055 mg/mL), **2** (0.076 mg/mL), **3** (0.073 mg/mL), **4** (0.073 mg/mL), **5** (0.063 mg/mL), **6** (0.057 mg/mL), **7** (0.061 mg/mL), **8** (0.067 mg/mL), and **9** (0.037 mg/mL) in acetonitrile/water (20/80, v/v)) or 1:10 diluted (spiking experiment 2) to a seed, a napin-rich sample, and a cruciferin-rich sample. Afterward, the samples were treated as described above. Additional unspiked samples were prepared as well.

#### Inter- and Intraday Precision

Three aliquots of the same rapeseed seed, napin-rich, and cruciferin-rich samples were measured for compounds **1**–**9** on the same and different days, yielding intra- and interday precision given as the relative standard deviation of the replicate analysis. Intraday: **1** (10%), **2** (8%), **3** (4%), **4** (11%), **5** (11%), **6** (8%), **7** (15%), **8** (6%), **9** (5%) and interday: **1** (12%), **2** (14%), **3** (12%), **4** (27%), **5** (24%), **6** (14%), **7** (42%), **8** (22%), **9** (58%).

### High-Performance Liquid Chromatography (HPLC)

The HPLC (Jasco, Groß-Umstadt, Germany) consists of a binary pump system PU-2087, a UV-2075 UV Detector, and a Rh 7725 injection valve (Rheodyne, Bensheim, Germany). For data acquisition, Chrompass Chromatography Data System version 1.9 was used.

### Liquid Chromatography-Mass Spectrometry (LC-MS/MS)

An AB Sciex 5500 Qtrap mass spectrometer (Sciex, Darmstadt, Germany) with direct flow infusion was used to acquire mass and product ion spectra. The instrument was controlled with Analyst 1.6.2 software (Applied Biosystems, Darmstadt, Germany) and was operated in full-scan mode (negative, ion spray voltage, −4500 V): curtain gas (35 psi); temperature (400 °C); gas 1 (45 psi); gas 2 (65 psi); collision-activated dissociation medium; and entrance potential (−10 V). Substances **1**–**9** and the **IS** were dissolved in acetonitrile/water and infused to give the specific product ions and ionization parameters (Table 2).

The MS system was connected with a Shimadzu Nexera X2 UHPLC system (Sciex, Darmstadt, Germany) consisting of a DGU-20A 5R degasser, two LC30AD pumps, a SIL30AC autosampler (kept at 15 °C), and a CTO30A column oven (40 °C). Separation of the substances was performed on a 100 mm × 2.1 mm i.d., 1.7 μm, Kinetex C18 100 Å (Phenomenex, Aschaffenburg, Germany) by injecting aliquots (2 μL) of the samples into the system running at a flow rate of 0.4 mL/min and using 0.1% formic acid in water and 0.1% formic acid in acetonitrile as solvents A and B, respectively. The following gradient was used: starting with 5% B, hold 5% for 3 min, increase in 2 min to 15% B, increase in 4 min to 30% B, increase in 1 min to 100% B, hold 100% for 2 min, decrease in 1 min to 5% B, and hold for 2 min isocratically.

### Nuclear Magnetic Resonance Spectrometry (NMR)

A 400 MHz DRX spectrometer and a 500 MHz Avance II spectrometer (Bruker, Rheinstetten, Germany) were used to record 1D and 2D NMR spectra. Samples were dissolved in D_2_O, DMSO-*d*_6_, ACN*-d*_3_, or methanol-*d*_4_ (600 μL), and chemical shifts were reported in parts per million (ppm) relative to solvent signals. Topspin NMR (Bruker) and MestReNova (Mestrelab Research, Santiago de Compostela, Spain) were used for data processing. Quantitative NMR data (q-NMR) was obtained via calibrating the spectrometer by applying the ERETIC 2 tool using PULCON methodology.^26^

### Statistical Analysis

The quantitative data were visualized as jittered faceted points plots using R (Version 4.0.2, R Foundation).^27^ Visualization was done using the package “ggplot2”.^28^ Significances for IPX determination and changes in gene expression were calculated according to an unpaired Student’s t-test with the software Graph Pad Prism 10.2.1.

### Cell Culture and Cell Viability

The human gastric tumor cell line HGT-1, obtained from Dr. C. Laboisse (Laboratory of Pathological Anatomy, Nantes, Frances), was used in the cell culture experiments. Cells were cultured in DMEM with 4 g/L glucose, supplemented with 10% fetal bovine serum, 3% l-glutamine, and 1% penicillin/streptomycin under standard conditions at 37 °C and 5% CO_2_.

Impaired cellular viability after treatment with **1** or **8** was excluded by means of an MTT assay as a measure of cellular proliferation. A total of 100,000 HGT-1 cells per well were seeded in 96-well plates 24 h prior to the viability test. The HGT-1 cells were exposed to the test substances for a total period of 30 min, The MTT solution was aspirated after 15 min of incubation at 37 °C. The purple formazan salt formed was dissolved in DMSO before measuring the absorption at 570 nm with a reference wavelength of 630 nm using a Tecan infinite M200 PRO plate reader (Tecan, Mannedorf, Switzerland).

### Determination of the Intracellular Proton Index (IPX) in HGT-1 Cells as an Outcome Measure of Proton Secretion Modulated by **1** and **8**

The intracellular pH, calculated as the intracellular proton index (IPX) as an indicator of cellular proton secretion linked to bitter taste receptor (TAS2R) regulation, was measured in HGT-1 cells by means of the pH-sensitive fluorescent dye SNARF-1-AM.^29^ Briefly, 100,000 HGT-1 cells were seeded in a black 96-well plate. After 24 h, cells were stained with 3 μM SNARF-1-AM for 30 min at standard cell culture conditions as detailed previously,^29^ and they were treated with either **1** or **8** for 10 min. Histamine (1 mM) was used as an internal reference, whereas HGT-1 cells exposed to KRHB only were used as a control.^29^ Fluorescence was measured at 580 and 640 nm emissions after excitation at 488 nm by means of a Flexstation 3 (Molecular Devices, San Jose, California). Using a nigericin calibration curve, the intracellular pH and the resulting intracellular H^+^ concentration were calculated. Hence, the ratio between treated and nontreated cells (KRHB only) was calculated, and log 2 was transformed to determine the intracellular proton index (IPX).

### Quantitation of mRNA Expression of Bitter Taste Receptors in HGT-1 Cells

A total of 1,000,000 viable HGT-1 cells were spread in a 6-well plate and allowed to settle for 24 h at 37 °C, 95% humidity, and 5% CO_2_. After incubation with either **1** (6.8 μm) or **8** (650 μM) for 30 min, RNA was isolated using the peqGOLD RNA kit. The quantity and quality of RNA were spectrophotometrically checked at a wavelength of 260 nm and by calculation of the absorbance ratio at 260 and 280 nm wavelengths using a NanoDrop One (Thermo Fisher Scientific Inc.). Removal of gDNA and synthesis of cDNA were performed using the iScript gDNA Clear cDNA Synthesis Kit following the manufacturer’s protocol. Real-time qPCR (RT-qPCR) was performed with 50 ng of cDNA amplified with Sso Advanced Universal SYBR Green Supermix. Peptidylprolyl isomerase A (PPIA) and glyceraldehyde 3-phosphate dehydrogenase (GAPDH) were used as reference genes.

## Results and Discussion

Although K3OSS (**1**) was recently found to be the key bitter tastant in rapeseed protein isolates,^10^ the following uncertainty arose: depending on the rapeseed source and the process used to obtain the isolates, additional kaempferol glycosides might contribute to the astringent and bitter taste impression in rapeseed isolates.

**Figure 2:**
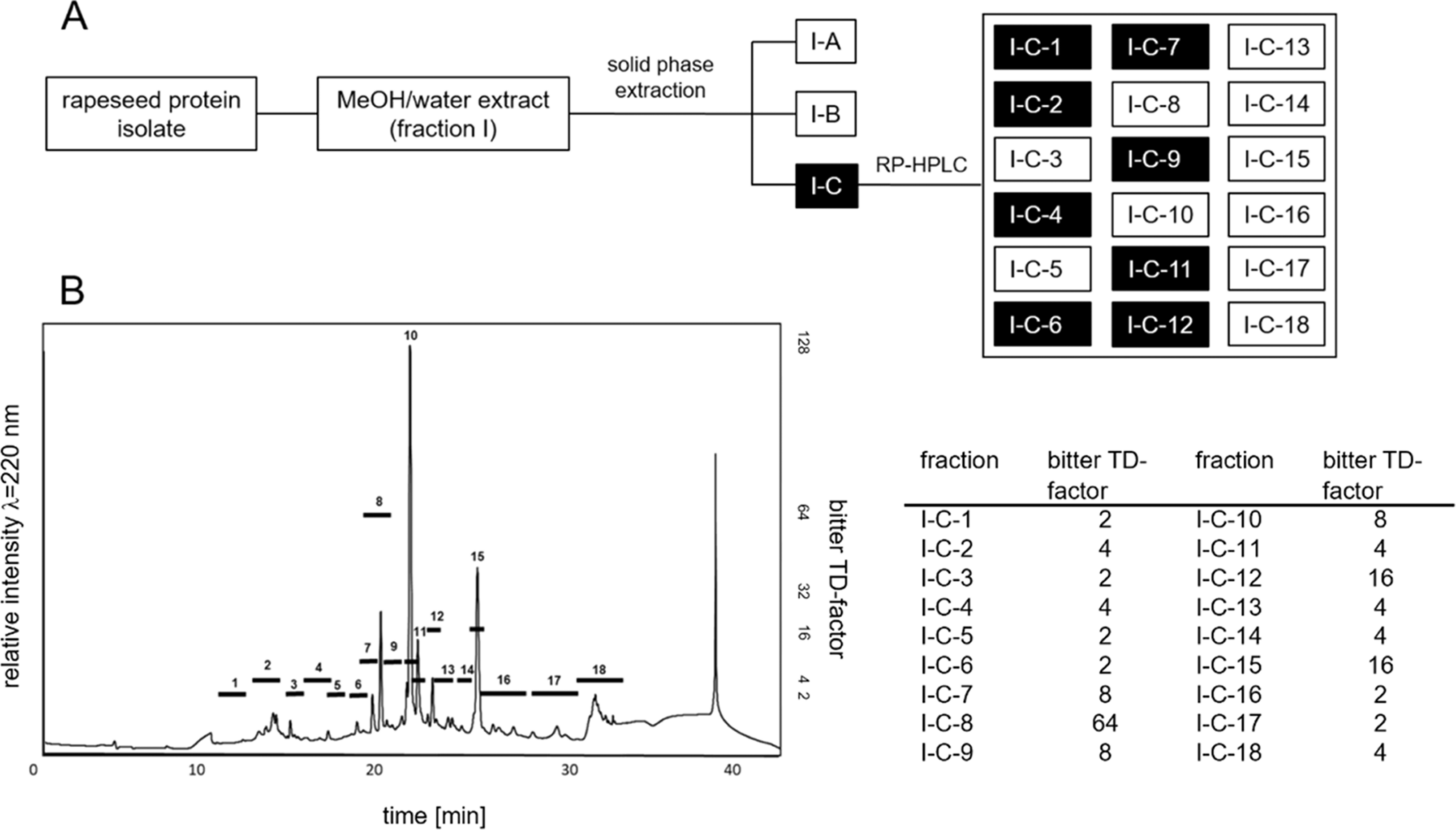
(A) Separation scheme used to locate bitter and astringent-tasting kaempferol glycosides and (B) RP-HPLC chromatogram and taste dilution (TD) factors of fraction I-C prepared from rapeseed protein isolate according to Hald et al.^10^

An activity-guided approach was recently applied to a methanol/water extract prepared from a rapeseed protein isolate following the fractionation strategy highlighted in Figure 2.^10^ As fraction I-C exhibited both the highest (i) bitter and astringent impressions as well as (ii) kaempferol glycoside contents measured by means of untargeted UHPLC-ToF-MS measurements, the following investigation was focused on the isolation and structure determination of the kaempferol glycosides in fractions I-C-1 to I-C-18.

### Isolation and Identification of Kaempferol Glycosides in HPLC Fractions I-C-1, -2, -4, -6, -7, -8, -9, -11, and -12

Fractions I-C-1 to I-C-18 (Figure 2) were screened via UHPLC-ToF-MS in the negative mode for additional potentially taste-active kaempferol glycosides by searching for the specific mass-to-charge ratio of the key fragment of the aglycone of kaempferol derivatives [aglycone-H]^−^ at 284 Da. Different kaempferol derivatives could be proposed in fractions I-C-1, -2, -4, -6, -7, -8, -9, -11, and -12.

Fraction I-C-8 contained the main bitter compound kaempferol 3-*O*-(2‴-*O*-sinapoyl-β-d-sophoroside) (**1**), as described in our previous paper.^10^

To identify the compounds in the corresponding fractions, they were further separated by means of semipreparative HPLC. After purification, their structures were elucidated by means of LC-ToF-MS, LC-MS/MS, partial hydrolysis, and NMR spectroscopy. In addition to the kaempferol component, glucose and sinapoyl moieties could be detected via specific fragmentation losses of 162 and 206 Da during the MS/MS measurements. To determine the intramolecular connection of those motifs, NMR spectroscopy experiments were performed. In the heteronuclear multiple-bond correlation spectroscopy (HMBC), the coupling between ^1^H and ^13^C atoms and therefore the connection of the sinapoyl, glucose, and kaempferol parts could be observed.

LC-MS (ESI^–^) analysis of compound no. **2**, isolated from fraction I-C-1, revealed *m*/*z* 771.2 as the pseudo-molecular ion ([M – H]^−^), thus suggesting a molecular mass of 772 Da. This was confirmed by LC-TOF-MS, indicating an empirical formula of C_33_H_40_O_21_. Additional LC-MS/MS experiments, performed in the ESI^–^ mode, led to the identification of the daughter ions *m*/*z* 446 [M-Glu-Glu-H]^−^, *m*/*z* 429 [M-H_2_O-Glu-Glu-H]^−^, and *m*/*z* 284 [M-Glu-Glu-Glu-H_2_O–H]^−^, thus demonstrating the presence of three hexose moieties in the target kaempferol glycoside. To further confirm the structure of the aglycone and to identify the sugar moieties, 1/2D NMR and hydrolysis experiments were performed.

The integrals of the signals in the ^1^H NMR spectrum of compound **2** displayed a total of 40 protons with signals resonating between 3.24 and 8.05 ppm. The proton signals observed between 6.52 and 8.05 ppm were assigned to the polyphenol protons of the kaempferol moiety. In addition, the ^1^H NMR spectrum of **2** displayed three anomeric sugar protons resonating at 4.86, 5.18, and 5.15 ppm. The coupling constant of the anomeric protons of *J*_1,2_ = 8.0 Hz specified a β-configuration. Due to the strong signal overlap of the sugar protons in ^1^H NMR experiments, the unequivocal identification of the monosaccharide type by NMR analysis was impossible. Therefore, the sugar building blocks were determined by acid hydrolysis and derivatization, followed by LC-MS/MS measurements in comparison to reference monosaccharides. The analysis afforded only d-glucose as sugar residues in the compound isolated from fraction I-C-1. Generally, in all analyzed compounds (**1** – **9**), only d-glucose could be determined by acid hydrolysis and LC-MS/MS.

To further confirm the structure of the aglycone and to identify the linkage positions of the sugar moieties, 2D NMR experiments were performed. In the HMBC experiment, the anomeric proton H–C(1″) as well as H–C(1*) showed a coupling to the carbon C(3) or C(7), resonating at 133.1 and 161.6 ppm, respectively. Additionally, a coupling between the anomeric atoms H–C(1‴) and C(2″) was observed, revealing a sophoroside moiety attached to C(3). This leads to the identification of kaempferol 3-*O*-β-d-sophoroside-7-*O*-β-d-glucopyranoside (**2**) in fraction I-C-1 with human recognition threshold concentrations of 184 and 4.8 μmol/L for the bitter and astringent perception, respectively (Table 1). Although **1** was identified earlier to be present in the leaves and seeds of *B. napus*, to the best of our knowledge, the presence of this kaempferol glycoside in rapeseed proteins as well as its bitter and astringent activity has not been reported.^14,15^

**Figure 3:**
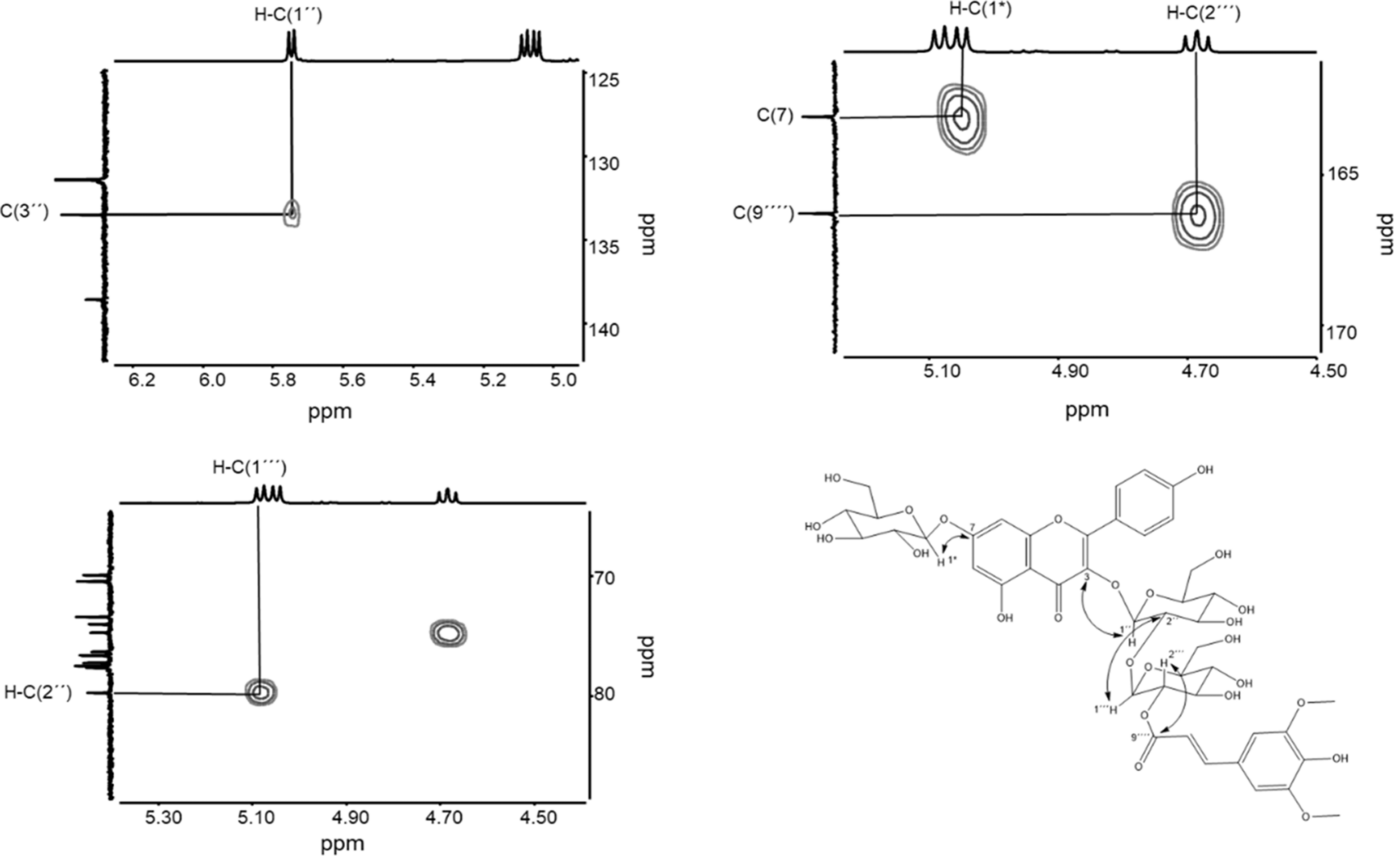
Excerpts of HMBC spectra (500 MHz, DMSO-*d*_6_) and chemical structure of kaempferol 3-*O*-(2‴-*O*-sinapoyl*-β*-d-sophoroside)-7-*O-β*-d-glucopyranoside (**3**).

LC-ToF-MS analysis of compound no. **3**, isolated from fraction I-C-2 showed a pseudo-molecular ion [M – H]^−^ with *m*/*z* of 977.2563. Additional MS/MS experiments in the ESI^–^ mode led to the identification of daughter ions with *m*/*z* 815 [M-H-Glc]^−^, 609 [M-H-Glc-Sinapoyl]^−^, 591 [M-H-Glc-Sinapoyl-H_2_O]^−^, 446 [M-H-Glc-Glc-Sinapoyl]^−^, 429 [M-H-Glc-Glc-Sinapoyl-H_2_O]^−^, and 284 [M-H-Glc-Glc-Glc-Sinapoyl-H_2_O]^−^, thus indicating the presence of three glucose and one sinapoyl moiety in the taste-active kaempferol glycoside. This finding was further confirmed by the identification of d-glucose in an acid hydrolysate by means of derivatization and UHPLC-MS/MS analysis. Comparing the proton spectra from fractions I-C-1 and -2, the same basic structure could be observed with an additional *E*-configured sinapoyl moiety. This residue showed a correlation between the anomeric proton H–C(1‴) of a sugar moiety and the carbon atom C(9‴′) of the carboxylic acid (Figure 3), leading to the identification of kaempferol 3-*O*-(2‴-*O*-sinapoyl-β-d-sophoroside)-7-*O*-β-d-glucopyranoside (**3**, Figure 1). This compound has been already identified as the most abundant kaempferol-glucoside in the leaves and seeds of *B. napus*.^14,15^ Its occurrence in rapeseed protein and its human recognition threshold of 160.7 for bitterness and 19.4 μmol/L for astringency have not been reported so far.

Compound no. **4** isolated from fraction I-C-4 showed the same *m*/*z* ratio in MS experiments as **3**, exhibiting the same elemental composition. However, the different retention times suggest an isomeric structure. ^1^H and ^13^C NMR spectra of **4** revealed the signals expected for a kaempferol aglycone, three glucose, and one sinapoylic acid moiety. Compared to compound no. **3**, heteronuclear correlation experiments revealed different linkage positions for the sugar moieties and the sinapoyl residue for tastant no. **4**. For example, the HMBC spectrum of **4** showed connectivities between the anomeric glucose protons H–C(1″) and H–C(1*) to the carbons C(3) and C(7), respectively. In addition, in the HMBC experiment, a coupling between the anomeric proton H–C(1‴) of the third glucose moiety and the C atom C(4′) could be observed. Furthermore, the ester carbon atom at 167.1 ppm [C(9‴′)] showed a coupling to the protons of the (*E*)-configured double bond [7.56 ppm of H–C(7‴′) and 6.56 ppm of H–C(8‴′)], as well as to H–C(6‴) of the sugar moiety. The identified compound kaempferol 4′-(6-*O*-sinapoyl-β-d-glucopyranoside)-3,7-di-*O*-β-d-glucopyranoside (**4**) was identified previously in the seeds of *B. napus*.^14,16^ But for the first time, this compound was identified as a bitter and astringent compound in rapeseed protein isolates, exhibiting human bitter and astringent taste thresholds of 320.8 and 11.5 μmol/L, respectively.

By comparing the HMBC spectra of fraction I-C-6 and kaempferol 3-*O*-β-d-sophoroside-7-*O*-β-d-glucopyranoside (**2**), the same correlation between H–C(1″) of the glucose moiety and C(3) as well as from H–C(1‴) to C(2″) were observed, confirming a sophoroside moiety at position C(3). This is well in line with the MS^2^ data showing a *m*/*z* ratio of 609, indicating that the kaempferol aglycone has only two glucose moieties and is lacking of the glucose attached at C(7) of the aglycon. The NMR data of kaempferol 3-*O*-β-d-sophoroside (**5**) identified in fraction I-C-6 were in agreement with those reported earlier, but this is the first time that **5** has been identified in rapeseed protein isolates with bitter and astringent taste thresholds of 531.7 and 66.4 μmol/L, respectively.^13^

Kaempferol 3-*O*-β-d-sophoroside-7-*O*-(2*-*O*-sinapoyl-β-d-glucopyranoside) (**6**) could be identified in fraction I-C-7, showing the same correlations between the sugar moieties and the kaempferol aglycone as kaempferol 3-*O*-β-d-sophoroside-7-*O*-β-d-glucopyranoside (**2**). The presence of a sinapinic acid was proposed by the MS^2^ spectrum, which exhibited a *m*/*z* of 977, indicating kaempferol, sinapinic acid, and three glucose units. Additionally, a coupling between H–C(2*) and the carbon C(9**) was observed, connecting the sinapinic acid moiety to the glucose via an ester. This compound has already been identified in rapeseed previously,^14^ but this is the first time that the full ^13^C NMR spectrum could be assigned, as well as the human taste threshold for bitterness (243.9 μmol/L) and astringency (35.1 μmol/L).

In fraction I-C-9, a *m*/*z* of 1183.3147 was detected by HR-MS, indicating kaempferol, two sinapoyl, and three glucose moieties. Homo- and heteronuclear correlation experiments gave a comprehensive picture of the type of moieties linked to kaempferol as well as on the conformation of the anomeric protons. For example, ^2,3^*J*_H,C_ correlations observed in the HMBC spectrum between the anomeric proton H–C(1″) and C(3) as well as H–C(1‴) and C(2″), confirmed the linkage between the kaempferol and sophoroside moiety. The ^2,3^*J*_H,C_ correlation between H–C(1*) and C(7) finally completes the connection of the sugar units. The linkage of sinapinic acids was determined by the correlation of the protons H–C(6*) or H–C(2‴) and the respective carboxylic acid carbon atoms C(9**) and C(9‴′). Taking all spectroscopic and spectrometric data into consideration, the compound isolated from fraction I-C-7 could be identified as kaempferol 3-O-(2‴-*O*-sinapoyl- β-sophoroside)-7-*O*-(6-*O*-sinapoyl-β-d-glucopyranoside) (**7**) with a bitter taste threshold of 265.1 and a recognizing astringency above 16.6 μmol/L. Although compound **7** has been identified in rapeseed previously,^13^ to the best of our knowledge, ^13^C NMR data and its taste activity have never been reported.

Substance **8**, exhibiting UV–visible (UV–vis) absorption maxima at 255 and 339 nm, showed a pseudo-molecular ion [M – H]^−^ with *m*/*z* 447 and a daughter ion with *m*/*z* 284 upon cleavage of a hexose moiety. By comparison of LC-MS and NMR data with those obtained from the literature, this astringent and bitter sensing compound eluting in fraction I-C-11 could be identified as 3-*O*-β-d-glucopyranoside (**8**).^17,30^ The substance exhibits a bitter taste above 324.7 and an astringent taste above 29.7 μmol/L.

Furthermore, the LC-ToF-MS analysis of fraction I-C-12 compared to compound **4** suggests the absence of one glucose moiety. In addition, the ^13^C NMR signals of the compound isolated from fraction I-C-12 showed that in comparison to **4** only the signals of the glucose unit at position C(7) were missing. Although the identified kaempferol 4′-(6-*O*-sinapoyl-β-d-glucopyranoside)-3-*O*-β-d-glucopyranoside (**9**) had already been described in rapeseed, the details of the ^13^C NMR shifts and the human bitter (149.3 μmol/L) and astringent (8.1 μmol/L) taste thresholds were not reported previously.^13^

In summary, nine kaempferol glycosides (**1**–**9**) were identified in fractions I-C-1, −2, −4, −6, −7, −8, – 9, −11, and −12 with human recognition thresholds of 3.4–531.7 μmol/kg for bitterness and 0.3–66.4 μmol/kg for astringency (Figure 1 and Table 1). Surprisingly, K3OSS (**1**) exhibited by far the lowest bitter and astringent recognition taste thresholds. Compound **5**, which exhibits the same sugar moieties as **1**, but lacks sinapinic acid, led to 100 times higher thresholds, implying the importance of sinapinic acid linked to position C(2‴) for the overall taste impression. Although **2**, **5**, and **8** were kaempferol glycosides without sinapinic acid esters, they still taste bitter and astringent. In addition, also the linkage position of the sugars influence the taste impression. For example, compared to **1** bitter stimuli **3**, bearing an additional sugar moiety at position C(7) of the aglycone showed 30 times higher thresholds, signaling the foundational importance of the linkage and amounts of sugars attached to the aglycone.

To decrease the off-taste of rapeseed proteins, different strategies can be applied.^31^ For example, the content of kaempferol glycosides can be decreased by applying breeding strategies targeting the kaempferol pathway, different postharvest technological downstreaming process steps, and enzymatic or fermentative approaches. Alternatively, the off-taste can be masked by adding bitterness inhibitors. To identify suitable inhibitor substances, the respective activated receptor needs to be determined.^31^

### Effect of Kaempferol 3-*O*-(2‴-*O*-Sinapoyl-β-d-sophoroside) (**1**) and Kaempferol 3-β-d-*O*-Glucopyranoside (**8**) on the Cellular Bitter Response in HGT-1 Cells

In the next step, we aimed to get first insights into a functional involvement of TAS2Rs in the bitter taste qualities of the isolated compounds and conducted a cellular TAS2R-dependent bitter response assay. For this assay, compounds **1** and **8** were selected according to their sensory bitter taste threshold concentrations. With compound **1**, for which a bitter taste threshold concentration of 3.4 μmol/kg was revealed, the most bitter-tasting compound identified was chosen. Compound no. **8** was identified as the second least bitter compound, with a bitter taste threshold concentration of 324.7 μmol/kg. Compound no. **8** was preferred over the least bitter-tasting compound no. **5** since compound no. **5** neither was commercially available nor to be isolated or synthesized in the amounts needed within a reasonable amount of time. To gain first insights on the molecular basis of the bitter taste of compounds **1** and **8**, the cellular bitter response of HGT-1 cells as a well-established cell model for the identification of bitter-tasting and bitter taste modulating compounds was studied.^29,32^ When treated with bitter-tasting compounds, HGT-1 cells respond by the secretion of protons, which is based on (1) upregulation and/or binding of the bitter-tasting compound to bitter taste receptors (TAS2Rs), followed by (2) the secretion of protons, which results in a lower intracellular proton concentration (calculated as intracellular proton index, IPX). To exclude the effects of kaempferol compounds **1** and **8** on the viability of HGT-1 cells, an MTT assay was performed. The tested concentrations of compounds **1** and **8** were chosen based on double bitter taste threshold concentrations revealed from sensory studies (Table 1). None of the compounds impaired the viability of HGT-1 cells compared to the corresponding control (KRHB).

Quantitation of the intracellular proton index (IPX) as a measure of cellular proton secretion in HGT-1 cells represents a well-established model for the identification of bitter-tasting and bitter taste modulating compounds targeting TAS2Rs.^29,33^ The IPX is quantitated by means of a pH-sensitive fluorescent dye that allows to calculate the secretion of protons according to changes of the IPX in untreated control cells vs treated cells.^29,33^ While negative IPX values resulting from treatments with bitter-tasting compounds represent a TAS2R-mediated increased proton secretion, thereby indicating an increased secretory activity as cellular bitter response, positive IPX values resulting from treatments with bitter-masking compounds represent a TAS2R-mediated antisecretory effect.^29,33^ In addition, changes in HGT-1 cells’ TAS2R mRNA levels have been recently demonstrated by our group to correlate well with IPX values and sensory bitter perception.^34,35^

**Figure 4:**
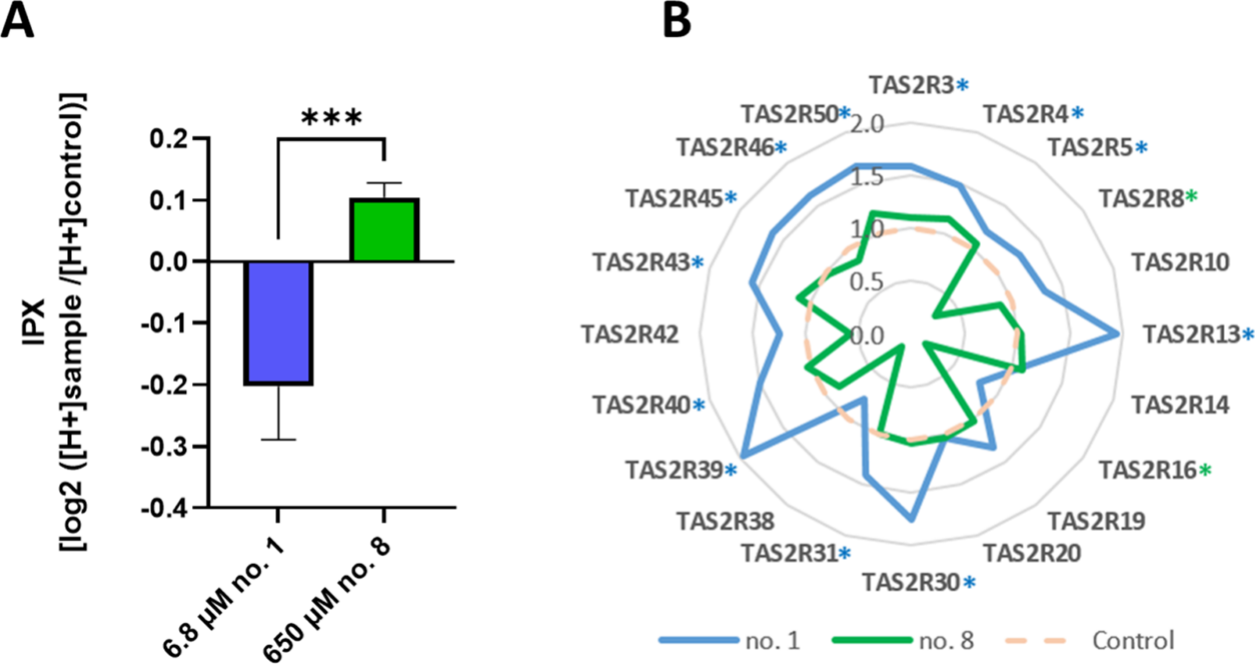
(A) IPX of HGT-1 cells after treatment with compound **1** or **8**, *n* = 4; six technical replicates (tr). Data presented as mean ± SEM. Statistics: unpaired *t* test. Significant (*p* < 0.05) differences are indicated as follows: ***p < 0.001. (B) Radar chart showing the changes in gene expressions (mRNA, fold change) of 20 bitter taste receptors (TAS2Rs) in HGT-1 cells after incubation for 30 min with **1** (6.8 μmol/L) or **8** (650 μmol/L). The results were normalized to the expression of PPIA and GAPDH (reference genes). Data are shown as mean, *n* = 4, tr = 3. Gene expression data for TAS2R1, R7, R9, R41, and R60 were excluded due to low expression (ct values > 38) in HGT-1 cells, either with or without treatment. The significance of gene regulation according to an unpaired *t* test is indicated by color-coded blue (compound no.1) and green (compound no. 8) stars.

In the sensory analysis, compound no. **1** was perceived as more bitter than compound no. **8** as the human taste recognition thresholds of compounds **1** and **8** were found to be 3.4 and 324.7 μmol/L, respectively. A similar result was obtained from the HGT-1 IPX analyses, where double taste threshold concentrations of 6.8 μmol/L for **1** elicited a stronger bitter response with an IPX of −0.2028 than 650 μmol/L of **8** with an IPX of 0.1040 (*p* = 0.0005; Figure 4). Additionally, the calculation of the AUC of the proton secretion over 30 min time resulted in a stimulation of proton secretion, indicated by negative IPX values, for compound **1** (AUC = −7.341), and in a reduced secretory activity, indicated by positive IPX values, for compound **8** (AUC = 5.776). Moreover, RT-qPCR analyses of TAS2Rs gene expression support these results since the exposure of HGT-1 cells to 6.8 μmol/L of compound no. **1** resulted in a more pronounced regulation of TAS2Rs compared to the cells treated with 650 μmol/L of compound no. **8** (Figure 4b). Specifically, mRNA expression of the bitter receptors TAS2R3, 4, 5, 13, 30, 31, 39, 40, 43, 45, 46, and 50 was regulated in HGT-1 cells treated with compound no. **1**. In comparison, only two TAS2Rs, namely, TAS2R8 and 16, were regulated after incubation of the HGT-1 cells with compound 8. Overall, the cell-based results clearly indicate TAS2Rs to be targeted more effectively by compound no. **1**, thereby explaining its lower bitter taste threshold compared to compound no. **8**.

### LC-MS/MS Method Development and Validation for Compounds (**1**–**9**)

**Figure 5:**
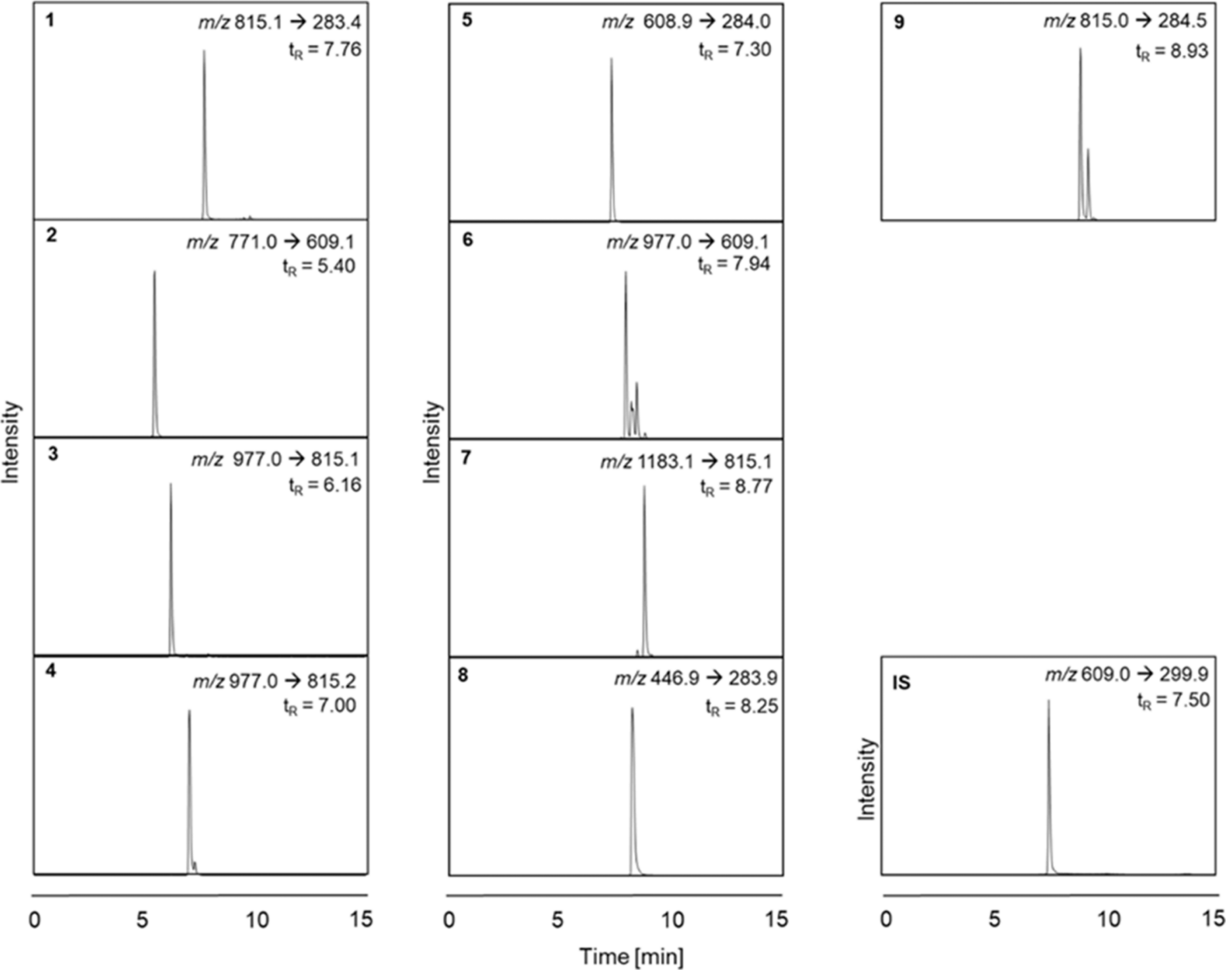
Mass transitions and retention times for the quantification of bitter compounds **1**–**9** as well as the **IS**.

To accurately analyze the target compounds **1**–**9** according to the SENSOMICS approach^31^ and thus determine the contribution of the isolated compounds to the off-taste in different rapeseed and rapeseed protein products, a quantification method was developed using rutin as **IS** because of its high structural similarity to the analytes. For each compound (**1**–**9**), specific MS/MS parameters were tuned in the negative ionization mode by directly infusing the compounds into the MS/MS system using a syringe pump. For sensitive quantification, the most abundant mass transitions were selected (Figure 5).

The analytes were extracted from rapeseed as well as from the corresponding rapeseed protein by using a mixture of MeOH/H_2_O and adding rutin as **IS**. The samples were simultaneously crushed and extracted, and after equilibration on a horizontal shaker, the samples were centrifuged. An aliquot of the supernatant was used for the LC-MS/MS measurement. Seeds and cruciferin-rich and napin-rich samples were spiked prior to the quantification with two different concentration levels of **1**–**9** to calculate the recovery rates. A comparison of the spiked samples with the natural samples revealed averaged recovery rates of 92% (**1**), 121% (**2**), 96% (**3**), 94% (**4**), 101% (**5**), 112% (**6**), 100% (**7**), 90% (**8**), and 108% (**9**) for the seed, 86% (**1**), 98% (**2**), 97% (**3**), 104% (**4**), 101% (**5**), 108% (**6**), 103% (**7**), 99% (**8**), and 112% (**9**) for the cruciferin-rich sample, and 96% (**1**), 130% (**2**), 114% (**3**), 106% (**4**), 120% (**5**), 86% (**6**), 85% (**7**), 102% (**8**), and 107% (**9**) for the napin-rich sample. The limit of detection was determined to be 0.22 and the limit of quantification was 0.68 μg/mL.

### Quantification of Taste-Active Compounds **1**–**9** in Rapeseed Protein Seeds and Isolates as well as Monitoring of Metabolic Changes during the Protein Production Process

**Figure 6:**
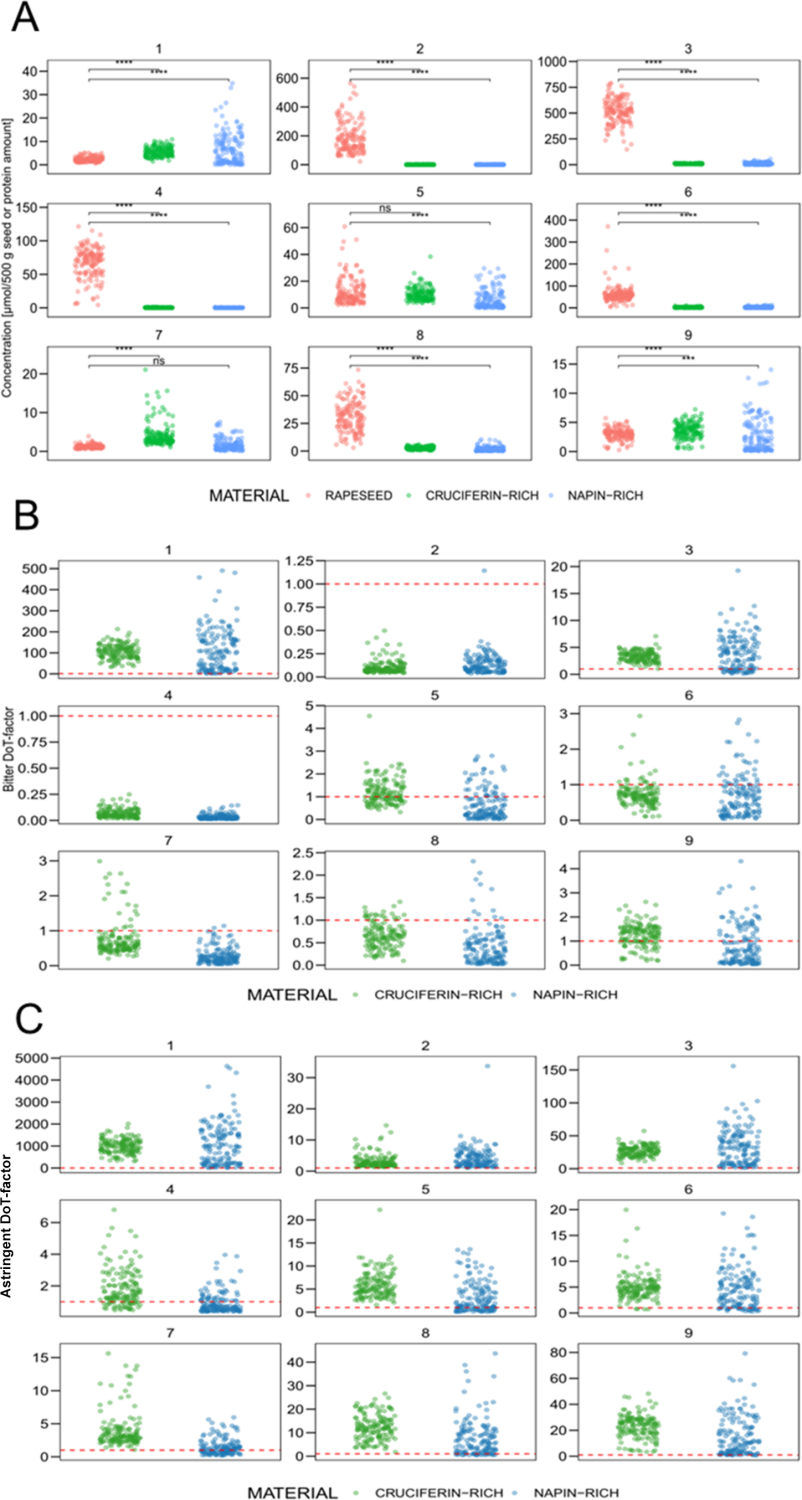
(A) Jittered points plot of quantitative data for compounds **1**–**9** in 150 rapeseeds and their respective cruciferin-rich and napin-rich rapeseed protein isolates. ns = not significant, *** = *p*-value < 0.005, **** = *p*-value < 0.0005 (Wilcox test). (B) Jittered points plot of bitter DoT factor for compounds **1**–**9** in 150 cruciferin-rich and napin-rich rapeseed protein isolates. (C) Jittered points plot of astringent DoT factor for compounds **1**–**9** in 150 cruciferin-rich and napin-rich rapeseed protein isolates.

The concentrations of compounds **1**–**9** of the rapeseed seed samples were normalized on 500 g of sample material, while the amounts for the protein were normalized on the amount of protein received from 500 g of rapeseed seed. The total amount of kaempferols is higher in the seeds than in the received proteins, ranging from 426 μmol/500 g to 1426 μmol/500 g, and compounds **2** and **3** are by far the most abundant in the rapeseed seed. During the protein extraction process, the amounts of compounds **2**–**4**, **6**, and **8** were significantly decreased in the protein samples compared to the initial rapeseed samples. At the same time, the amounts of compounds **1**, **7**, and **9** increased (Figure 6). With the exception of the glucose at position C(7), compounds **1** and **3** as well as **4** and **9** indicate structural similarity. Since the amounts of **3** and **4** decreased and the amounts of **1** and **9** increased, the idea arises that during protein production, enzymatic activity and/or chemical hydrolysis may lead to the liberation of glucose from position C(7). Due to their similar chemical features and based on these quantitative data, **3** could be identified as a possible precursor to liberating **1** during protein processing. Cleavage of the respective sugar moiety leads to the presence of substances with a lower bitter and astringent taste threshold (Table 1); therefore, in the case of **1** and **3**, it will lead to a more bitter-tasting product as the human recognition threshold of **1** is at least 40 times lower than for the other compounds. Consequently, even a small amount of **1** will dramatically enhance the overall bitter taste of rapeseed proteins.

To assess the bitter and astringent taste activity of compounds **1**–**9** in cruciferin- and napin-rich rapeseed protein isolates, Dose over Threshold (DoT) factors were determined as a ratio of the concentration of the respective tastant to the taste threshold.^36^ Depending on the 150 measured samples, both cruciferin- and napin-rich rapeseed protein isolates exhibited DoT factors calculated for bitterness ≥ 1 for compounds **1**, **3**, and **5**–**9**. Comparing the dose-overthreshold (DoT) factors of kaempferol glycosides (Figure 6), **1** shows the highest impact on bitter taste with DoT factors up to 480. Conversely, compounds **3** and **5**–**9** only sometimes exceed DoT factors above one.

In contrast to bitterness, the astringency of all other kaempferol glycosides seems to contribute to the off-taste of rapeseed proteins (Figure 6C) as their DoT factors are above one. Compounds **3**, **8**, and **9**, in particular, exhibit higher values for astringency and might influence taste perception, while **2**, **4**, **5**, **6**, and **7** most likely only slightly contribute to the overall taste.

In summary, the receptor studies, as well as the quantification data, reveal the importance of **1** to the overall bitter taste of rapeseed proteins. Compound **1** had for example a higher response in the receptor tests compared to kaempferol 3-*O*-β-d-glucopyranoside (**8**) and a lower human bitter taste threshold. Furthermore, it accumulates during the protein isolation process formed from precursor kaempferol glycoside **3**, which could not completely be removed during the protein isolation process. In addition, this study demonstrated for the first time that compounds **1**–**9** noncovalently binding to rapeseed proteins contribute to the overall unpleasant astringency of rapeseed protein isolates.

These results can contribute to the production of less bitter and astringent-tasting rapeseed and canola protein isolates through the optimization of breeding, masking, and postharvest downstream processes. Additionally, we hypothesize that added selective enzymes could hydrolyze the kaempferol glycosides, which could be analyzed by the developed method.^31^
